# A Lecture on Hæmoptysis

**Published:** 1839-02-02

**Authors:** N. Chapman

**Affiliations:** Professor of the Theory and Practice of Physic, in the University of Pennsylvania


### MEDICAL EXAMINER.

DEVOTED TO MEDICINE, SURGERY, AND THE COLLATERAL SCIENCES.

No. 5.] PHILADELPHIA, SATURDAY, FEBRUARY 2, 1839. [Vol. II.

### A LECTURE ON HÆMOPTYSIS.

*By* N. Chapman, M. D., *Professor of the Theory and Practice of Physic, in the University of Pennsylvania.*

(Continued from page 55.)

The mucous membrane throughout its distribution in the lungs, their appendages, as well as in the fauces, being liable to a sanguineous exhalation, it may be useful to distinguish the immediate position of it. Generally, when the hæmorrhage proceeds from the latter structures, there is merely hawking without any pulmonary oppression, cough, or vascular excitement,—and, on examination of the throat, we shall, in some instances, perceive the source itself whence the blood comes.

As to hæmoptysis proper, it is, perhaps, impossible always to discriminate accurately, by symptoms only, its two forms—that of the mucous membrane, and of the tissue of the lungs themselves. Being, however, a *pulmonary* case, by which I mean an affection of the lung itself, it is usually designated by all those characteristics I have just mentioned as belonging to the most violent attacks of the affection, coming on more suddenly, and with such intense oppression as even to threaten suffocation.

Nor can we uniformly rely on the external means of exploration,—though, by carefully weighing the respective indications, much may be learnt. In hæmorrhage of the mucous membrane, the chest, on percussion, is perfectly sonorous, and the stethescope betrays a mucous rattle, proportionate to the quantity of blood retained in the bronchiæ. The former of these means in the pulmonary engorgement, when it is considerable, elicits a dull sound over the affected part,—and the latter shows a want of the respiratory murmur in it, and the crepitous, instead of the mucous rattle. Cases, however, are to be met with of great ambiguity, and where these resources fail, as when the two affections are united, or the engorgement is slight, or is seated in a portion of the lungs beyond the reach of percussion. Taken in any view, the decision of the point is rather a matter of curiosity than practical utility. But very different is it in regard to the chronic lesions, as tubercles, hepatization, &c., of which the effusion may be the effect, and happily as it is important, so is it comparatively easy. He who is skilled in the application of these means, is enabled, at once, to pronounce with tolerable certainty, on the nature of the affection, and to institute the practice best fitted for its removal.

It may be affirmed as a general proposition, that the inactive is far more intractable than the active hæmoptysis. There are, however, other considerations by which the case is to be estimated in this respect. The degree of danger is influenced by the position whence the hæmorrhage proceeds, and still more so by the pathological condition with which it may be associated. Coming from the larynx and adjacent structures, it has been deemed the least alarming, and which may be generally so, though sometimes of the most serious import, even such as are apparently confined to the fauces. But it is presumable, that the larynx was at the time, or subsequently became involved,—and when hæmorrhages from this structure are precursory to consumption, as they are apt to be, probably the lungs were previously affected, the morbid action ascending upwards, or, as may be, the reverse, the irritation descending till the whole pulmonary system became engaged. Caused by tubercles, or any other organic lesion, an unfavourable result is sooner or later to be anticipated,—and such as emanate from the pulmonary substance itself, are almost uniformly and speedily fatal. The latter, however, are happily of rare occurrence. Belonging to the mucous membrane, on the contrary, copious as may be the effusion, little is directly to be apprehended. Death, indeed, from hæmoptysis, as an immediate consequence, very seldom happens. Heberden informs us, that in a practice of sixty years, he never lost a patient by it,—and my own experience, which extends to two-thirds of this period, supplies me with very few instances—and none of these, I have reason to believe, were of the mucous membrane. Three of the four fatal cases which I have seen, were, indeed, by a post-mortem examination, shown not to be so. The vicarious, or metatastic hæmorrhage, is still less to be regarded.

For the most part, hæmoptysis ought to carry with it little further terror than that excited by a suspicion, too often well-founded, that it is an outward sign or expression of disease of the lungs, and especially a tubercular state of these organs. Exempt from such lesions, they seem, in some instances, to suffer no more from hæmorrhage, than other parts,—in proof of which, I have several very striking facts supplied by my own observations. Cases have come under my care, where the hæmorrhage had been repeated again and again for a series of years, which ultimately did well,—and there is no reason to doubt, that a very distinguished person of this city, who died not long ago in his ninetieth year, was subject to very frequent recurrences of it, for nearly two-thirds of his life. Yet, though the lungs may escape from any serious injury, it were prudent in every case to effect a cure. The event itself shows an undue irritation in these organs,—for, without it, the afflux of the blood leading to the effusion would not take place,—and it is obvious, that this is the very state of things, which, by continuance, is so apt to lay the foundation of irreparable mischief.

Considering the diversity of condition on which hæmoptysis depends, the post-mortem appearances must, of course, be expected to vary. From the infrequency of death in that of the mucous membrane, the phenomena are not precisely determined. But we are not without some intelligence regarding them, and the following has been reported. In the most simple cases, caused merely by turgescency of the vessels of the tissue, little is observable, excepting the surface is covered more or less with blood—the congestion having been relieved by the previous effusion. Connected with actual phlogosis, the ordinary evidence of such a state in the mucous lining is exhibited,—and, being a chronic case, there are some changes of structure, thickened, and either softer, or more indurated, or condensed, than natural, with, occasionally, fibrinous concretions in the form of polypi.

Extravasations taking place in the parenchyma, which, as I said, is a very rare event, the appearances resemble very much those in cerebral apoplexy. Looking at the lung thus affected, we shall discover such portions of it circumscribed, from one to two or three inches, of a very deep dark red colour, and of a density equal to the completest hepatization. Cutting into these portions, they are found to consist of concrete blood—the surrounding tissue being crepitant, and of the usual colour, or reddish, as if tinged with blood.

These are the phenomena in the simplest states of hæmoptysis. But in the more complicated forms of it, there are discoverable, in different instances, besides tubercles in their several stages of developement, all those organic lesions to which the lungs are exposed. Extending, too, our researches further, we may find the heart, the liver, spleen, or other of the viscera, variously diseased, while the lungs shall escape or not, the irritation causing the effusion of blood being entirely of a derivative nature in the former case.

(To be continued.)

